# Using mobile virtual reality to enhance medical comprehension and satisfaction in patients and their families

**DOI:** 10.1007/s40037-019-0504-7

**Published:** 2019-03-25

**Authors:** Adam Palanica, Michael J. Docktor, Andrew Lee, Yan Fossat

**Affiliations:** 1 Labs Department, Klick Health, Klick Inc., Toronto, ON Canada; 2 Division of Gastroenterology, Boston Children’s Hospital, Boston, MA USA

**Keywords:** Digital medicine, Precision medicine, Virtual reality, Patient engagement, Medical education

## Abstract

Patients are typically debriefed by their healthcare provider after any medical procedure or surgery to discuss their findings and any next steps involving medication or treatment instructions. However, without any medical or scientific background knowledge, it can feel overwhelming and esoteric for a patient to listen to a physician describe a complex operation. Instead, providing patients with engaging visuals and a virtual reality (VR) simulation of their individual clinical findings could lead to more effective transfer of medical knowledge and comprehension of treatment information. A newly developed VR technology is described, called HealthVoyager, which is designed to help facilitate this knowledge transfer between physicians and patients. The platform represents a customizable, VR software system utilizing a smartphone or tablet computer to portray personalized surgical or procedural findings as well as representations of normal anatomy. The use of such technology for eliciting medical understanding and patient satisfaction can have many practical and clinical applications for a variety of disease states and patient populations.

## Introduction

It is estimated that over 310 million medical operations occur worldwide each year [1]. After nearly all surgical and medical procedures, patients are typically debriefed by their physician to discuss their findings and any next steps involving care at home or treatment instructions. Unfortunately, medical information is complex and time consuming to properly convey to patients and their families. Patients are typically provided generic handouts and documents intended for the medical record to illustrate what are often unique conditions that are difficult for them to comprehend. Adding to the problem are the demands on clinicians who are increasingly unable to dedicate the necessary time to this important process.

Unfortunately for patients and their families, studies have shown that people recall as little as half of what is discussed during a typical medical encounter [2]. Research has shown that 40–60% of patients could not correctly report what their physicians expected of them 10–80 min after they were provided with information, and other research demonstrated that over 60% of patients interviewed immediately after visiting their doctors misunderstood the directions regarding prescribed medications [3]. This may be due to patients’ lack of understanding of their disease and lack of involvement in the treatment decision-making process, placing them at risk for increased rates of hospitalization and poorer clinical outcomes [4]. In the US alone, an estimated 90 million adults have inadequate health literacy, meaning that they have difficulty understanding complex text and acting upon health information [5]. Another problem that may occur during medical encounters is that physicians may fail to communicate all medical information to their patients. One study found that in 66% of audiotaped cases analyzed, physicians had omitted at least one piece of critical information when discussing a new medication with a patient [6].

Comprehension of medical information may also be adversely affected when patients are debriefed on their operation by physicians who are not their primary healthcare provider, which can lead to updated information being lost in the communication between the patient, proceduralist, and providers. In all of these instances, it is likely that critical information may not be properly communicated to the patient [6]. Furthermore, the nature of the presented information is usually complex, and it is often difficult for the healthcare provider to accurately gauge a patient or family member’s level of understanding in the brief time available in the postoperative setting. Without any medical or scientific background knowledge, it can feel overwhelming and esoteric for a patient to listen to a physician describe a complex operation. This can result in a negative impact on patient satisfaction, the quality of the physician-patient relationship, the likelihood of treatment adherence, and patient health outcomes [7]. These barriers to communication may be further exacerbated in the paediatric population where the patient can be less medically literate, less engaged in the material, or less aware of their condition.

## Presenting personalized medical information

Most physicians share procedural findings with the patients and their families using clinical documents that are highly text-based, written in medical language, some with static thumbnail images, and are designed for medical documentation, but not necessarily for patient understanding. Most adults, and especially children, have a limited attention span, and cannot process loads of complex information given at once, especially when it is presented in text-based esoteric language. Too much or complex information can lead to cognitive overload which can tax the ability to process and remember the message being conveyed [8]. Additionally, patients may be repulsed by the graphic medical images of internal anatomy.

Thus, using different ways of presenting information can influence how well it is attended and understood, and can also affect how motivated people are to respond to that information. For example, instead of overloading the patient with text-based content, an alternative option is to break-up the data with more personalized information and animated visuals to improve the patient’s attention to important information. Presenting information that is vivid, concrete, and personalized is an effective way to increase attention and persuasiveness [9]. Personalized information can be more salient, and is more likely to be remembered and lead to changes in future behaviour [10]. Additionally, displaying information visually can improve the understanding of that information by creating context for better memory. Simple visual cues and illustrations can attract attention, and are often less cognitively taxing than text-based information [11].

## Presenting medical information with virtual reality

An alternative to presenting medical information using traditional methods is with interactive multimedia technologies, such as virtual reality (VR) and techniques adapted from the video game industry. VR and gamification methods have been shown to be an effective education training tool to supplement in-person education and competency assessment [12]. VR is able to provide patients with feedback about their condition and, through game-like scenarios, can increase the overall motivation and engagement during education [13, 14]. This interactive technology can improve health knowledge and assist in health-related decision making for the general population [15]. Interactive VR technology and gamification methods have also been especially beneficial for children to improve their overall education and comprehension of new ideas, and to increase their motivation and interest for learning [16–18].

Consequently, a virtual simulation that recreates a life-like scenario for patients and their families could lead to more effective transfer of medical knowledge and comprehension of treatment information regarding their surgical or medical procedures. In turn, this could save long-term physician consultation costs and improve the overall health of the patient.

## Introducing HealthVoyager

To overcome the disadvantages of delivering medical information via traditional methods, a proposed solution is to employ an immersive VR experience for increasing patient engagement, education, and understanding of their own disease to enhance patient satisfaction and adherence to treatment. As a result, the development of ‘HealthVoyager’ represents a customizable VR software platform utilizing a smart phone or electronic tablet to visually captivate patients and their families with an animated digital avatar of their own anatomy and clinical findings [19]. Developed in collaboration between Klick Inc. and Boston Children’s Hospital, the platform portrays personalized surgical or procedural findings as well as representations of normal anatomy for patients to better comprehend complex medical information. This technology allows physicians to create a customized digital character with the specific procedural findings of the patient which can then be viewed during debriefing, follow-up appointments, as well as at home using a patient-specific mobile application.

HealthVoyager was designed to overcome the challenges associated with delivering effective information during clinical debriefings by: 1) presenting more visual rather than text-based data, 2) delivering personalized rather than generic information, 3) using interactive rather than passive education methods, and 4) allowing patients to view their clinical findings at home at any time to review detailed information.

**Fig. 1:**
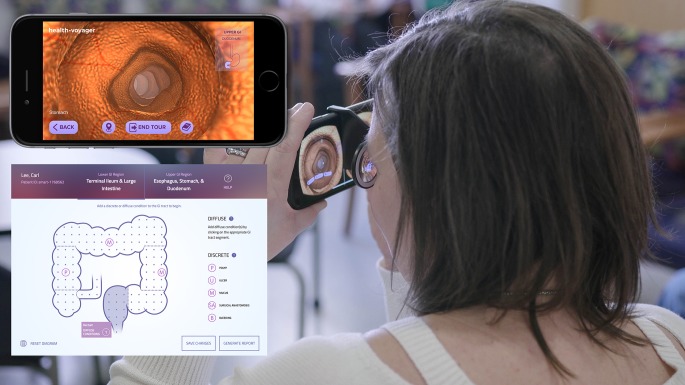
HealthVoyager software application, with an inlay of the patient VR experience (*top left*) and a sample report from the physician’s notes (*bottom left*)

The first iteration of the tool, HealthVoyager GI (Fig. 1), is designed for paediatric gastrointestinal (GI) patients and is currently in use at Boston Children’s Hospital to help enhance paediatric patient and family understanding, engagement, and satisfaction. By integrating HealthVoyager GI into the clinical workflow of endoscopic procedures, gastroenterologists at Boston Children’s Hospital can configure custom, lifelike, 3D anatomical imagery to show patients with conditions such as Crohn’s disease and ulcerative colitis a virtual tour of their GI tract. This personalized tour of one’s own GI tract can be reviewed in person with a high fidelity VR headset or tablet, and at home via a smartphone and Google Cardboard compatible case or accessory.

Boston Children’s Hospital performs thousands of endoscopic procedures each year, and understood the challenges involved in the patient journey when receiving complex medical information. They ideated on possible enhancements to improve patient satisfaction and knowledge comprehension. Additionally, Klick Inc. has expertise in developing interactive technologies within the healthcare field, and includes a team of professional medical illustrators who are specialized in anatomical 3D visualization. Klick also has previous relationships with Boston Children’s Hospital, and thus, a mutual partnership was created, which leveraged each party’s representative expertise in bringing HealthVoyager to reality. VR was chosen as a viable solution to enhance patient satisfaction and knowledge comprehension due to the large amount of literature supporting its effectiveness in healthcare education. Usability testing was also conducted on the first iterations of the platform to develop a finished product that works effectively in the environment of a fast-paced hospital setting.

**Fig. 2:**
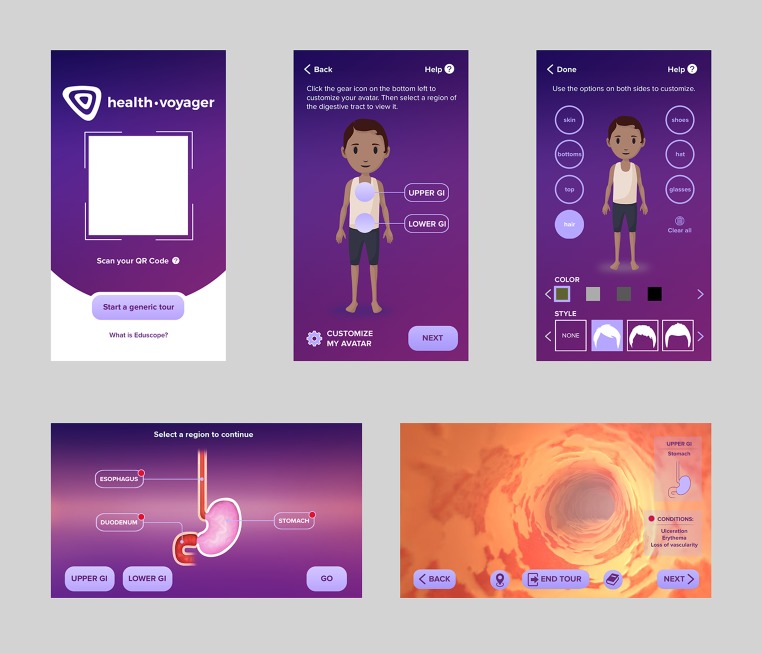
HealthVoyager patient-focused application interface with personalized avatar creation

HealthVoyager GI consists of three components: 1) a clinician-focused front-end interface to generate custom patient procedure reports, 2) a patient-focused mobile application that utilizes the individualized data for each patient, and 3) an intermediary, secure web service that ties the two together. Using a proprietary web interface, with drag-and-drop interactivity, the physician can precisely place the clinical findings of a patient’s endoscopy or colonoscopy onto digital illustrations of the upper or lower GI tract. This includes a list of various representations of both discrete clinical findings (e. g., polyps, ulcers, mucus, bleeding, surgical anastomosis, etc.) and diffuse clinical findings (e. g., erythema, granularity, loss of vascularity, ulceration, oedema, etc.) for the clinician to select. The physician creates the patient report, which takes about 2 min on average, and the tool automatically generates a unique de-identified patient Quick Response (QR) code, within seconds, to share with the patient and family. The patient then scans the custom QR code using their mobile phone to create their personalized avatar on the app, and access their personalized VR experience (Fig. 2). The experience is entirely interactive and patients can navigate in both directions across their entire GI tract simulation, while spending as much time as they want in each section, and view their full 360° surroundings. Patients can also go back to any section of the anatomy for free viewing exploration. One limitation is that users can only view the virtual surroundings, but not actually manipulate the environment (i. e., anatomy) since these represent the internal findings of their body.

The platform is designed to be accessible from within a hospital’s electronic medical record (EMR) system to protect the privacy and security of patient health information and ensure clinical adoption and sustainability. Additionally, the system can be deployed outside of the EMR environment but does not contain personal health information to remain compliant with Health Insurance Portability and Accountability Act regulations.

HealthVoyager GI is currently being evaluated for usability by both patients and clinicians at Boston Children’s Hospital. Preliminary qualitative data from 15 child patients and their parents, as well as two clinicians, were evaluated to assess satisfaction with using the platform. The clinician-focused front-end interface went through multiple iterations of user experience testing in order to achieve an efficient platform to input patient results in under 2 min on average. The resulting patient-focused mobile application was received with favourable enthusiasm by both patients and their parents. All patients felt more knowledgeable on learning about the GI anatomy, and felt that they could explain the findings to other friends or family. Patients felt informed during their debriefing, and felt that the physician’s report was very understandable. Overall, patients and parents were satisfied with their experience, and most individuals also stated that they were likely to use HealthVoyager again and would recommend the app if they knew another family needing a similar procedure.

Over time, it is expected that the more children and their families can visualize and understand their disease, the more satisfied they will become, and the more likely they will be to communicate when they have a particular symptom and to adhere to their therapies. Although the current application is examining paediatric patients with GI disorders, this platform can easily be expanded to other various diseases, anatomical areas, or patient populations.

## Conclusions

HealthVoyager offers a form of *precision medicine* (i. e., the customization of healthcare with medical diagnosis, treatments, and practices being tailored to individual patients) [20], and aims to create personalized educational experiences for patients and their families. By helping patients visualize and better understand their disease, this technology can be a more engaging and valuable way to learn about and share complicated medical information. This innovative system allows patients and their family members to actively explore representations of their own anatomy and procedural findings, rather than being passively told about their operation; they can spend as much time as they want learning about precise medical information in a fun and exciting way using VR and gamification methods.

Providing patients with a better understanding of their medical conditions should engage and empower them to become more active in sustaining their own health. This is especially important in the case of chronic childhood illnesses where adherence to a long-term medical treatment plan is vital. Enhancing medical comprehension and satisfaction in child patients and their parents should, in turn, allow physicians to better care for these individuals by strengthening the communication during all medical encounters. Using this form of precision education to enhance patient knowledge can be an important step towards increasing overall patient health and well-being.
